# The anti-microbial peptide TP359 attenuates inflammation in human lung cells infected with *Pseudomonas aeruginosa* via TLR5 and MAPK pathways

**DOI:** 10.1371/journal.pone.0176640

**Published:** 2017-05-03

**Authors:** Ejovwoke F. Dosunmu, Robert O. Emeh, Saurabh Dixit, Mona K. Bakeer, Mamie T. Coats, Donald R. Owen, Shreekumar R. Pillai, Shree R. Singh, Vida A. Dennis

**Affiliations:** 1 Center for NanoBiotechnology Research, Alabama State University, Montgomery, Alabama, United States of America; 2 Lousiana State University Health Sciences Center, School of Allied Health Professions, New Orleans, Louisiana, United States of America; 3 Therapeutic Peptides Inc., Baton Rouge, Louisiana, United States of America; Consejo Nacional de Investigaciones Cientificas y Tecnicas, ARGENTINA

## Abstract

*Pseudomonas aeruginosa* infection induces vigorous inflammatory mediators secreted by epithelial cells, which do not necessarily eradicate the pathogen. Nonetheless, it reduces lung function due to significant airway damage, most importantly in cystic fibrosis patients. Recently, we published that TP359, a proprietary cationic peptide had potent bactericidal effects against *P*. *aeruginosa*, which were mediated by down-regulating its outer membrane biogenesis genes. Herein, we hypothesized that TP359 bactericidal effects could also serve to regulate *P*. *aeruginosa*-induced lung inflammation. We explored this hypothesis by infecting human A549 lung cells with live *P*. *aeruginosa* non-isogenic, mucoid and non-mucoid strains and assessed the capacity of TP359 to regulate the levels of elicited TNFα, IL-6 and IL-8 inflammatory cytokines. In all instances, the mucoid strain elicited higher concentrations of cytokines in comparison to the non-mucoid strain, and TP359 dose-dependently down-regulated their respective levels, suggesting its regulation of lung inflammation. Surprisingly, *P*. *aeruginosa* flagellin, and not its lipopolysaccharide moiety, was the primary inducer of inflammatory cytokines in lung cells, which were similarly down-regulated by TP359. Blocking of TLR5, the putative flagellin receptor, completely abrogated the capacity of infected lung cells to secrete cytokines, underscoring that TP359 regulates inflammation via the TLR5-dependent signaling pathway. Downstream pathway-specific inhibition studies further revealed that the MAPK pathway, essentially p38 and JNK are necessary for induction of *P*. *aeruginosa* elicited inflammatory cytokines and their down-regulation by TP359. Collectively, our data provides evidence to support exploring the relevancy of TP359 as an anti-microbial and anti-inflammatory agent against *P*. *aeruginosa* for clinical applications.

## Introduction

In the lungs, ciliated epithelial cells play a major role in its defense against pathogens, by secreting chemokines (Keratinocyte Chemoattractant; KC) and cytokines (IL-6 and TNFα) [1], particularly the human neutrophil attractant, IL-8 [2]. In order for the host to initiate these responses, certain conserved microbial structures, pathogen-associated molecular patterns (PAMPs), have to be recognized by the host cell, and this occurs upon activation of toll-like receptors (TLRs) for induction of innate immune responses to phagocytose and kill the pathogen. This is obtained by the interaction between the microbial pathogen and the epithelial cells lining the alveolar surface and mammalian airways [3]. However, in immunocompromised hosts, such as cystic fibrosis (CF) patients, the bacterial pathogen is not readily eradicated resulting in an exaggerated immune response.

*Pseudomonas aeruginosa* infection induces vigorous inflammatory mediators [4, 5] such as IL-8, IL-6 and TNFα, which are secreted by epithelial cells through cell signaling pathways [2], and which do not necessarily eradicate the pathogen. When in excess, they cause decreased lung function due to significant airway damage. Specifically, in CF patients [6], chronic lung infections with *P*. *aeruginosa* and its associated inflammation are a major cause of morbidity and mortality [7]. The non-mucoid (NMPA) variant of *P*. *aeruginosa* is the predominant phenotype during the establishment of infection; thereafter, there is a shift to a more persistent mucoid (MPA); variant [8]. This phenotype conversion results from the synthesis of a large quantity of alginate exopolysaccharide [9], which is preceded by the formation of protected biofilm micro-colonies [10]. *P*. *aeruginosa* expresses numerous PAMPs [11] including lipopolysaccharides (LPS) [12] and flagellin [13]. LPS is a glycolipid that constitutes the outermost membrane of Gram-negative bacteria [14], while flagellin is a protein that form the filament bacterial flagellum [15]. These PAMPs are sensed by encoded receptors called pattern recognition receptors (PRRs), that include TLRs, for example TLR4 and TLR5 that recognize LPS and flagellin, respectively and can initiate protective responses against *P*. *aeruginosa* infection. The importance of TLR4 and TLR5 in response to *P*. *aeruginosa* infection is illustrated by similar survival of singly deficient TLR4 or TLR5 mice as compared to their wild type controls after infection with *P*. *aeruginosa* strain PAK, and as opposed to reduced survival of TLR4 and TLR5 double knockout mice [1].

Anti-microbial peptides (AMPs) are molecules produced by cells of many tissues in animals, plants, and invertebrates; they are ancient host defense molecules present in a wide variety of organisms [16–18]. AMPs consist of a variety of amino acids and are characterized by their size, sequence, net charge, structure, hydrophobicity and amphipathicity [19]. Cationic AMPs possess abundant positively charged amino acids, such as arginine (R) and lysine (K) [16]. The positive charge on AMPs enables their antibacterial activity, because the attraction between positively charged AMPs and the negatively charged head group of some phospholipids in the bacterial outer membrane, such as phosphatylglycerol (PG) and cardiolipin, or LPS and teichoic acid, is the first step for exerting antibacterial activity, followed by the interaction, insertion, and membrane perturbation [20].

In the present study, we employed a proprietary peptide, TP359, which we recently showed to have potent bactericidal effects against *P*. *aeruginosa* [21], and the human A549 lung cells as a model system for studying *P*. *aeruginosa*-induced lung inflammation. Our hypothesis is that TP359 bactericidal effects could also serve to regulate *P*. *aeruginosa*-induced lung inflammation. First, we investigated the bactericidal effects of the peptide on human A549 lungs cells infected with live *P*. *aeruginosa* non-isogenic, mucoid and non-mucoid strains by quantification of the bacterial burdens. Second, using cytokine ELISAs, we determined the regulatory effects of TP359 on lung inflammation by quantifying TNFα, IL-6, IL-8 and IL-1β secretions in supernatants of A549 cells exposed to live *P*. *aeruginosa* strains. Third, we deciphered which *P*. *aeruginosa* PAMP is responsible for eliciting inflammatory responses in lung cells by focusing on its LPS and flagellin. Fourth, we specifically determined the major target of TP359 regulatory effects by performing antibody neutralization experiments, respectively for the LPS and flagellin putative TLR4 and TLR5 receptors. Lastly, we determined the downstream signaling pathways for cytokine induction in A549 cells infected with *P*. *aeruginosa* and the ensuing effect of TP359 on pathways, including p38, JNK, ERK as well as NF-kB. The results from our study are presented and discussed in this manuscript.

## Materials and methods

### Tissue culture

Human A549 lung epithelial cells ATCC^®^ CCL-185^™^ (American Type Culture Collection, Manassas, VA) were cultured in F-12K Medium (Life technologies, Grand Island, NY) supplemented with 10% fetal bovine serum (FBS; Life technologies) with or without 1% 100× antibiotic-antimycotic (Life technologies). Cell suspensions were either plated onto 12- or 24-well tissue culture plates (BD Falcon, Bedford, MA) or on 8-well culture chambers (BD Falcon) depending on the specific experiment. All cell cultures were incubated at 37°C in a 5% CO_2_ humidified atmosphere.

### Bacterial cultures and stimulants

We used *P*. *aeruginosa* non-isogenic, mucoid (ATCC 39324) and non-mucoid (ATCC 27318) strains for all studies as recently described [21]. Briefly, strains were grown overnight in Luria-Bertani medium (LB; Becton Dickson) at 37°C with shaking. The next day, 50 μL of overnight culture was added to 2 mL LB media and allowed to grow to mid-log phase, before adjusting suspensions to an optical density at 600 nm of 0.1 (1× 10^9^ CFU/mL). Bacterial suspensions for infection were prepared by pelleting them at 450× g for 10 minutes, washing them 2× with sterile phosphate-buffer saline (PBS), followed by re-suspension of bacteria to the same volume in culture medium without antibiotics. Bacteria were further diluted from 10^9^ to 10^8^ CFU/mL depending on the specific experiment. Quantitative analysis of bacterial numbers was determined by 100-fold serial dilutions, before plating on LB agar plates. Plates were incubated overnight at 37°C and bacteria were enumerated the following day.

*P*. *aeruginosa* flagellin (FLA) (Invivogen, San Diego, CA) was dissolved in water at a concentration of 0.2 mg/mL and stored at −80°C. *P*. *aeruginosa* LPS (Sigma-Aldrich, St Louis, MO) was used by dissolving the lyophilized powder in water at 1 mg/mL and stored at 4°C. Both FLA and LPS were diluted in antibiotic-free culture media to their optimum concentrations as predetermined in this study.

### Establishment of cell infectivity

To determine the multiplicity of infection (MOI), A549 lung cells (1 × 10^5^/well/mL) were plated in 24-well plates for 24 h to permit attachment. The next day, A549 cells were washed with antibiotics-free media, and prepared bacteria suspensions in 1 mL of antibiotics-free media were added at MOIs of 0.1, 1, 10 and 100 and incubated for 5 h at 37°C in a humidified atmosphere with 5% CO_2_. Consequently, to determine the optimum number of A549 cells and duration of infection required for inducing optimum concentrations of inflammatory mediators, varying A549 cell numbers (10^4^ and 10^5^ cells/well/mL) and (10^6^ cells/well/mL) were plated for 24 h in 24-and 12-well plates, respectively. The next day, A549 cells were washed with antibiotics-free media, and prepared bacteria suspensions in 1 mL of antibiotics-free media were added at desired MOI (100) at time-intervals of 1, 2, 3, 4 and 5 h.

For establishment of infectivity, A549 cells (10^4^ to 10^6^/mL) were plated in 24- and 12-well plates for 24 h. The next day, A549 cells were washed with antibiotics-free media, and prepared bacteria suspensions (10^6^ to 10^8^ CFU/mL) in 1 mL of antibiotic-free media were added, and incubated at 37°C for 5 h.

For all cell infectivity studies, cell-free supernatants were collected after duration of infection, and centrifuged at 450× g for 10 minutes before being filtered through 0.2-μm pore size syringe filter to remove any bacteria and then stored at −80°C until used.

### TP359 treatment

The effect of TP359 on regulating essential *P*. *aeruginosa*-elicited lung pro-inflammatory cytokines (IL-6, IL-8 and TNFα) was evaluated by plating A549 cells (1 × 10^6^ cells/well/mL) in 12-well plates overnight followed by infection for 4 h with live bacteria (MOI of 100). We selected this MOI and time-point since both induced optimum levels of the above mentioned pro-inflammatory cytokines. Three different strategies were employed to assess the effect of varying concentrations of TP359 (12.5, 25, 50 and 100 μg/mL) to regulate lung inflammation induced by either strain of *P*. *aeruginosa*. They include I) exposing TP359 and *P*. *aeruginosa* simultaneously to A549 cells, II) exposing A549 cells to TP359 for 1 h before infecting them with *P*. *aeruginosa*, and III) infecting A549 cells with *P*. *aeruginosa* for 1 h before exposing them to TP359. These strategies were employed to determine the optimum scenario, if any, for TP359 to regulate *P*. *aeruginosa*-elicited lung pro-inflammatory cytokines. Polymyxin B (PB) was included as a positive control in all experiments. Culture supernatants from all experiments were collected and filtered as described above and stored at −80°C until used. The TP359 concentration-dependent study was based on cell viability assay results as recently published, which demonstrated non-toxicity of TP359 to A549 lung cells up to a concentration of 200 μg/mL [21].

### Stimulation of A549 lung cells by FLA and LPS

Experiments were conducted to determine which *P*. *aeruginosa* moiety, notably FLA and LPS, triggers production of inflammatory cytokines in A549 cells and how inflammation may be regulated by TP359. The optimum concentrations of purified *P*. *aeruginosa* FLA and LPS used were determined by exposing A549 cells to varying concentrations of FLA and LPS (1–1000 ng/mL). FLA at 100 ng/mL and LPS at 1000 ng/mL secreted optimum concentrations of IL-6 (data not shown); hence these concentrations were used for subsequent experiments. A549 cells (1 × 10^6^/mL) were stimulated with FLA (100 ng/mL) or LPS (1 μg/mL) in 12-well plates and incubated for 4 h at 37°C in a humidified atmosphere with 5% CO2. A549 cells were infected with the mucoid strain to serve as a positive control. Unstimulated A549 cells served as negative control for all experiments. Cell-free cultured supernatants were collected and stored at −80°C until used.

### TLR4 and TLR5 neutralization

We conducted antibody neutralization experiments to determine the precise role played by FLA or LPS in the induction of pro-inflammatory cytokines by *P*. *aeruginosa*, and their inhibition by TP359. A549 cells (1 × 10^6^ cells/well/mL) were plated in 12-well plates for 24 h followed by exposure to pre-determined optimum concentrations of anti-TLR4 (10 μg/mL), anti-TLR5 (10 μg/mL) and their corresponding isotype control antibodies (Invivogen, San Diego CA) for 24 h. Bacteria suspensions (1 × 10^8^ cells), FLA (100 ng/mL) and LPS (1 μg/mL) in antibiotic-free media were then added and incubated for an additional 4 h at 37°C in 5% CO_2_. Cell-free cultured supernatants were collected and stored at −80°C until used.

### MAPK pathway inhibition

To further determine the downstream pathway employed by *P*. *aeruginosa* to trigger inflammatory mediators in human A549 lung cells, we conducted pathway inhibition studies using inhibitors for p38, JNK, NF-κB and ERK signaling pathways. We plated A549 cells (1 × 10^6^ cells/well/mL) in 12-well plates for 24 h and then exposed them for 24 h to varying concentrations (2.5–20 μM) of pathway-specific inhibitors: p38 MAPK (SB203580), ERK (U0126), JNKs XVI (JNK-IN-8) and NF-κB (activation inhibitor IV) all from EMD (Millipore, Billerica, MA). After 24 h incubation, bacteria (MOI of 100) were added and incubated for 4 h at 37°C in 5% CO_2_. Cell-free supernatants were collected, filtered and stored at −80°C until used.

### Western blot

A549 cells (5 × 10^6^ cells) were seeded in 60 mm × 15 mm cell culture dishes and infected with live *P*. *aeruginosa* (5 × 10^8^ CFU/mL) for 15, 30 and 60 minutes. Cells were lysed at different time-points using cell lysis buffer (Cell Signaling Technology, Danvers, MA) supplemented with 1 mM PMSF and phosphatase inhibitor (200 mM each of Na_3_VO_4_ and NaF) (Thermo Scientific, Wilmington, Delaware). Cells were transferred to microcentrifuge tubes, sonicated for 15 sec to shear DNA and reduce viscosity, followed by centrifugation at 12,000× g for 10 min at 4°C. The concentrations of proteins were determined by the bicinchoninic acid assay (BCA) (Thermo Scientific, Rockford, IL, USA). Proteins were separated by SDS-PAGE, transferred to nitrocellulose membranes, and blocked with blocking buffer [Tris-Buffered Saline (TBS) containing 0.1% Tween-20 and 5% w/v nonfat milk]. After blocking for 1 h, the membrane was washed 3 times for 5 min each with wash buffer (TBS, 0.1% Tween-20) and incubated overnight with gentle agitation at 4°C with phospho-p38 or total p38 primary antibodies (Cell Signaling Technology, Danvers, MA) each at a dilution of 1:1000 diluted in primary antibody dilution buffer (1× TBS, 0.1% Tween-20, 5% nonfat dry milk). Following overnight incubation, the membrane was washed 3 times and incubated for 1 h at room temperature with HRP-conjugated secondary antibody (Cell Signaling) at 1:2000 (diluted in blocking buffer) with gentile agitation. The membrane was washed 3 times and protein bands were visualized using LumiGLO substrate (Cell Signaling) on scientific imaging film (Kodak Inc., Rochester, NY, USA). The sizes of total p38 and phospho-p38 were determined from the biotinylated protein ladder. For other experiments, A549 cells were infected with *P*. *aeruginosa* (MOI of 100), FLA (100 ng/mL) and LPS (1 μg/mL) in the presence and absence of TP359 (100 μg/mL) to determine if TP359 may exert its anti-inflammatory activity by blocking the p38 MAPK pathway. Total p38 was used as internal control to verify protein loading.

### Statistical analysis

All data are expressed as the mean ± SD of samples run in triplicates from three independent experiments. Data were analyzed by Sigmaplot data analysis software using one-way ANOVA. The number of asterisk was based on the degree of significance such as *P < 0.05, **P < 0.01 and ***P < 0.001.

## Results

### *P*. *aeruginosa* mucoid strain induced more IL-6 than the non-mucoid strain in a cell-density and time-kinetics manner

**Fig 1:**
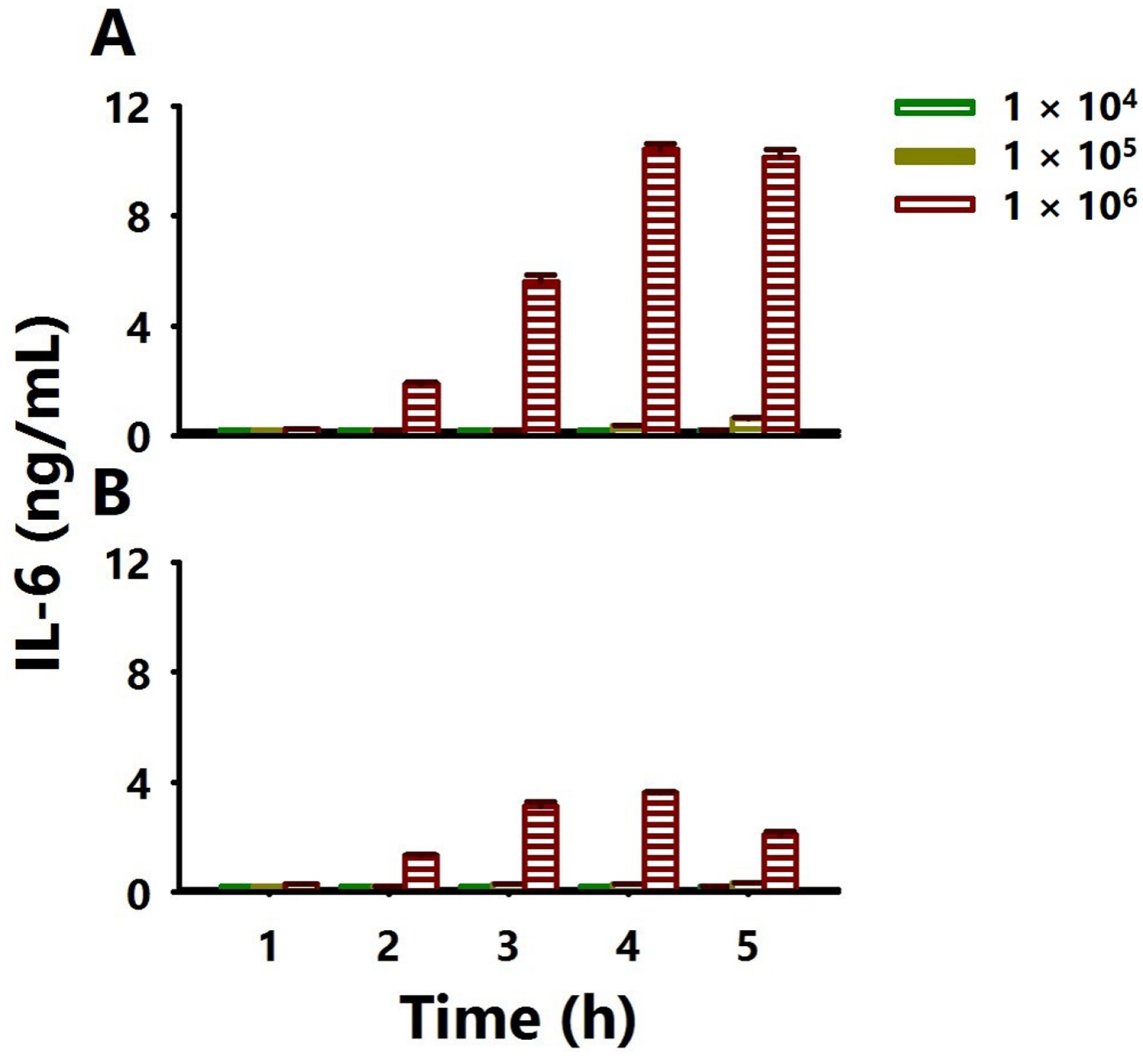
Optimum secretion of the IL-6 pro-inflammatory cytokine by *P*. *aeruginosa*-infected A549 cells in a cell-density and time-dependent kinetics. Varying A549 cell numbers (10^4^, 10^5^ and 10^6^ cells/well) plated in 24- and 12- well plates were incubated at 37°C in a 5% CO_2_ humidified atmosphere for 24 h. [A] mucoid and [B] non-mucoid *P*. *aeruginosa* strains (pre-determined optimum MOIs of 100) were then added and incubated at time-intervals of 1, 2, 3, 4 and 5 h. Cell-and bacteria-free culture supernatants were collected at each time-point to quantify IL-6 by specific ELISA.

Airway neutrophils of CF patients have been shown to have higher concentrations of pro-inflammatory cytokines compared to healthy patients, in response to *P*. *aeruginosa* infection [22]. Increased levels of IL-6, IL-8, TNFα and IL-1β have also been shown to be the prototypic cytokines of the CF airways [23]. Hence, we first selected IL-6 as a marker of inflammation to determine its secretion from *P*. *aeruginosa*-infected human A549 lung cells. To determine the optimum numbers of cells and duration of infection, different concentration of A549 cells were infected with either strain of *P*. *aeruginosa* at an MOI of 100. A549 cells infected with either strain of bacteria secreted high levels of IL-6 in a cell-density and time-kinetics manner (Fig 1). However, A549 cells infected with the mucoid strain secreted higher concentrations of IL-6 (Fig 1A) as compared to the non-mucoid strain (Fig 1B). Because the highest concentration of IL-6 was obtained at an A549 cell number of 1 × 10^6^ and at a 4 h time-point, these parameters were used in all subsequent experiments.

### TP359 dose-dependently reduced the production levels of multiple pro-inflammatory cytokines in *P*. *aeruginosa*-infected A549 cells

**Fig 2:**
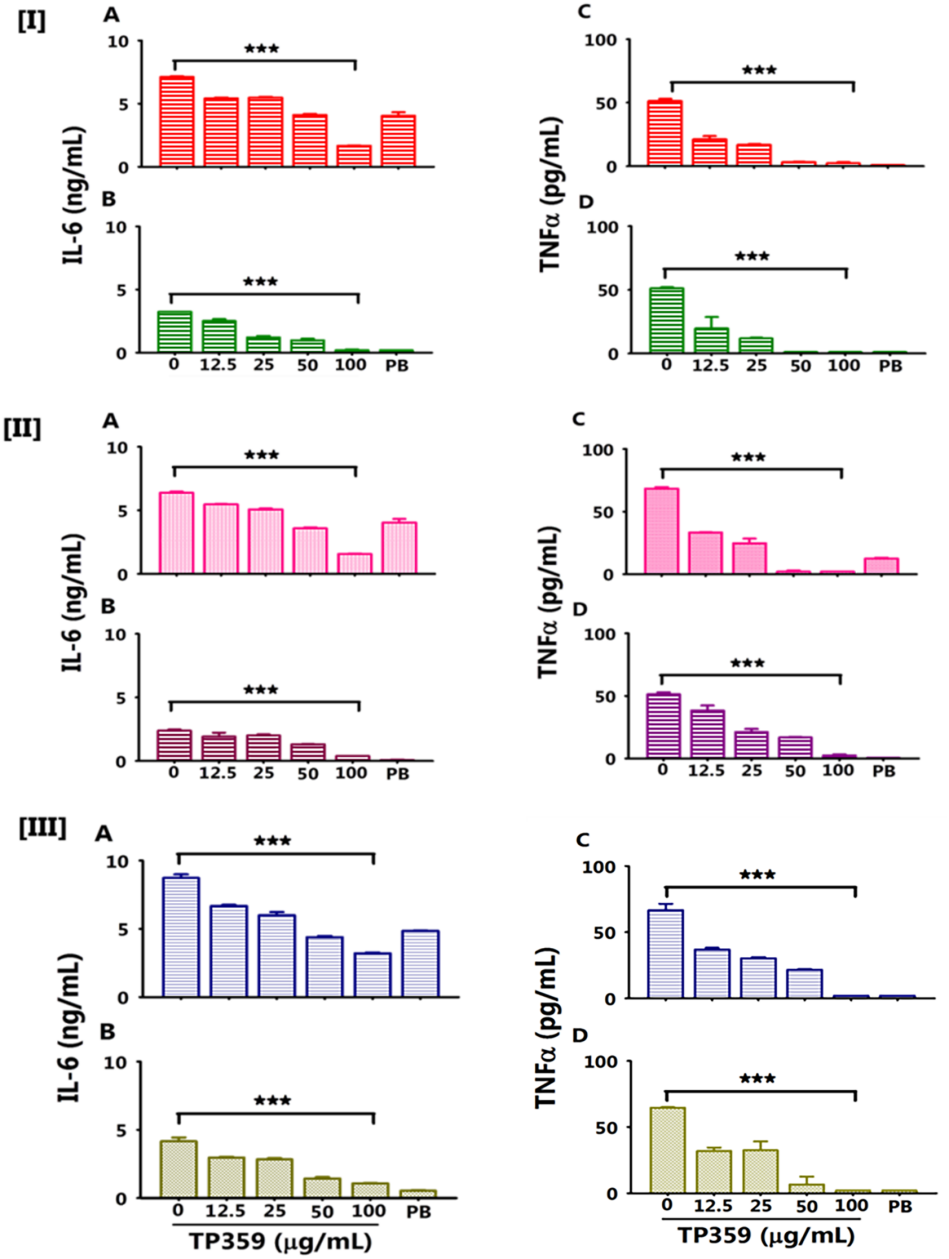
TP359 dose-dependently reduced the production levels of pro-inflammatory cytokines in *P*. *aeruginosa*-infected A549 cells. A549 cells (1 × 10^6^ cells/well/mL) were cultured in 12-well plates overnight followed by bacteria infection (MOI of 100) for 4 h. A549 cells infected with mucoid [A, C] or non-mucoid [B, D] strains of *P*. *aeruginosa* were then exposed to TP359 at various concentration (12.5, 25, 50 and 100 μg/mL) using three different strategies: [I] Exposing TP359 and bacteria simultaneously to A549 cells; [II] Exposing A549 cells to TP359 for 1 h, before bacterial infection; [III] Infecting cells with bacteria for 1 h, before exposing them to TP359. Positive control consisted of polymyxin (PB)-treated A549 cells infected with *P*. *aeruginosa*. Cell-and bacteria-free culture supernatants were collected to quantify cytokines by specific ELISAs. [A] and [B] are IL-6 concentrations, while [C] and [D] are TNFα concentrations, at each strategy for the mucoid- and non-mucoid *P*. *aeruginosa*-infected A549 cells. Each bar represents the mean ± SD of samples run in triplicates. The data are representative of three separate experiments. Statistical analysis was carried out using the one-way ANOVA. The number of asterisk was based on the degree of significance such as *P < 0.05, **P < 0.01 and ***P <0.001.

**Fig 3:**
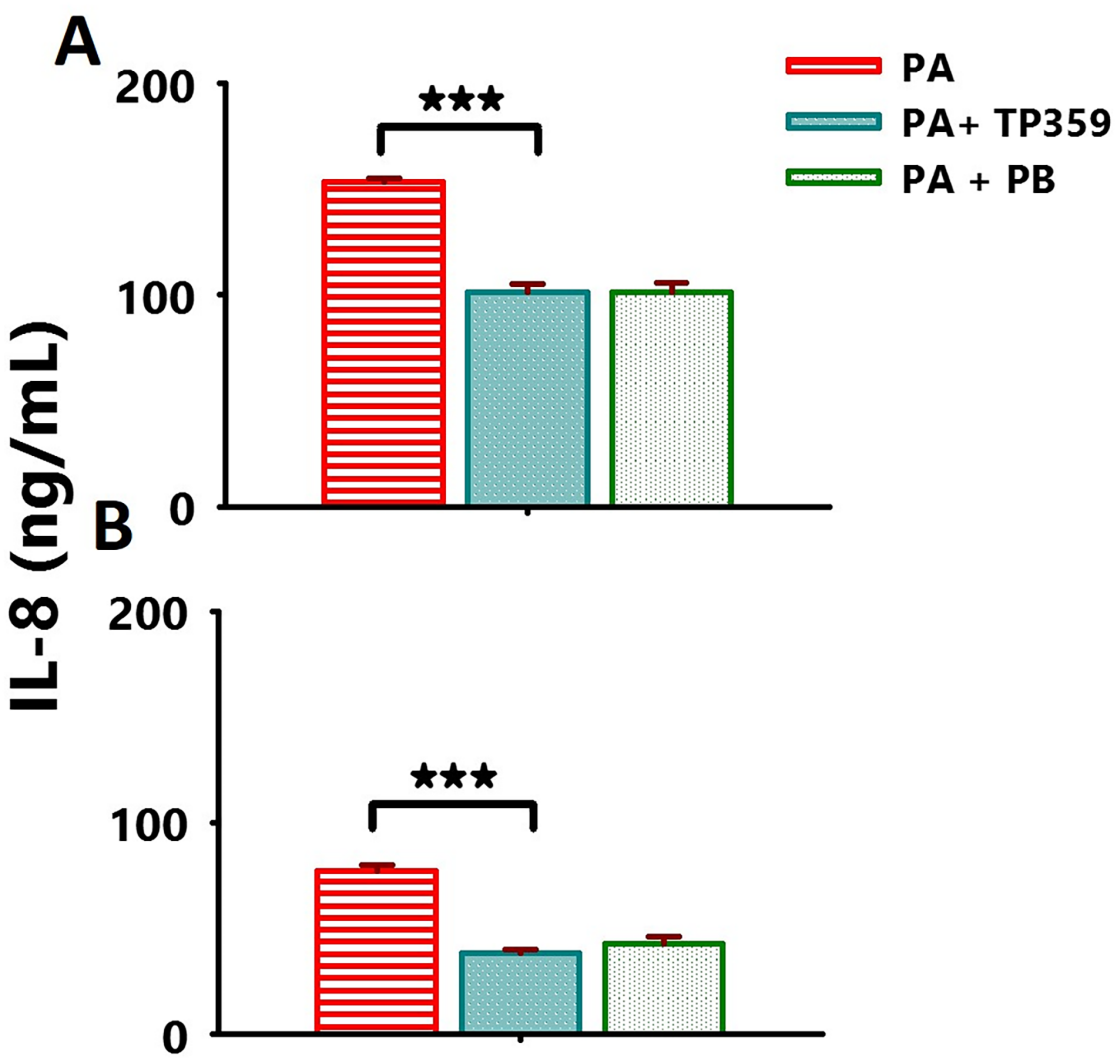
TP359 dose-dependently reduced the production levels of the IL-8 pro-inflammatory cytokine in *P*. *aeruginosa*-infected A549 cells. A549 cells (1 × 10^6^ cells/well/mL) were cultured in 12-well plates overnight followed by bacteria infection (MOI of 100) for 4 h. A549 cells infected with either [A] mucoid and [B] non-mucoid strains of *P*. *aeruginosa* were then exposed to TP359 at 100 μg/mL. Positive control consisted of polymyxin (PB)-treated A549 cells infected with *P*. *aeruginosa*. Cell-and bacteria-free culture supernatants were collected to quantify IL-8 by specific ELISA. Each bar represents the mean ± SD of samples run in triplicates. The data are representative of three separate experiments. Statistical analysis was carried out using the one-way ANOVA. The number of asterisk was based on the degree of significance such as *P < 0.05, **P < 0.01 and ***P <0.001.

We recently reported that TP359 has anti-microbial properties against *P*. *aeruginosa* mucoid and non-mucoid strains [21]. As such, we tested whether TP359 anti-microbial effect potentially could regulate the levels of pro-inflammatory cytokines produced by *P*. *aeruginosa*-infected A549 cells. A549 cells were exposed to either strain of *P*. *aeruginosa* in the presence and absence of varying concentrations of TP359 (12.5–100 μg/mL) followed by quantifications of IL-6 and TNFα. We used three different strategies to evaluate the putative anti-inflammatory effect of TP359 as described in the materials and methods section above. TP359 significantly (*P*<0.001) and dose-dependently inhibited the secretion levels of IL-6 (Fig 2A and 2B) and TNFα (Fig 2C and 2D), respectively for both bacteria strains and each strategy (I, II, III). In the first strategy [I], bacteria and TP359 were exposed to A549 simultaneously to determine the immediate effect of TP359 on A549 exposed to *P*. *aeruginosa*. In the second strategy [II], A549 cells were exposed to TP359 for 1 h to determine its possible use as a prophylaxis. In the third strategy [III], A549 cells were infected with *P*. *aeruginosa* for 1 h before being exposed to TP359 to determine the effect of TP359 after infection. TP359 (at 100 μg/mL) showed the best anti-inflammatory effect in all three strategies without any significant differences between strategies. Similar observations were made for IL-8 secreted by A549 cells infected with the mucoid strain and treated with TP359 (at 100 μg/mL) (Fig 3). Unstimulated cells did not secrete any cytokines (data not shown); however, PB-treated A549 cells exposed to the mucoid strain secreted both IL-6 and IL-8. These findings demonstrate that TP359 has anti-inflammatory properties against multiple pro-inflammatory cytokines, especially those that play a role in *P*. *aeruginosa*-induced inflammation. IL-1β concentration was under the detection limits for all three strategies (data not shown).

### The capacity of TP359 to reduce pro-inflammatory cytokines in *P*. *aeruginosa*-infected cells is partly due to its anti-microbial properties

**Fig 4:**
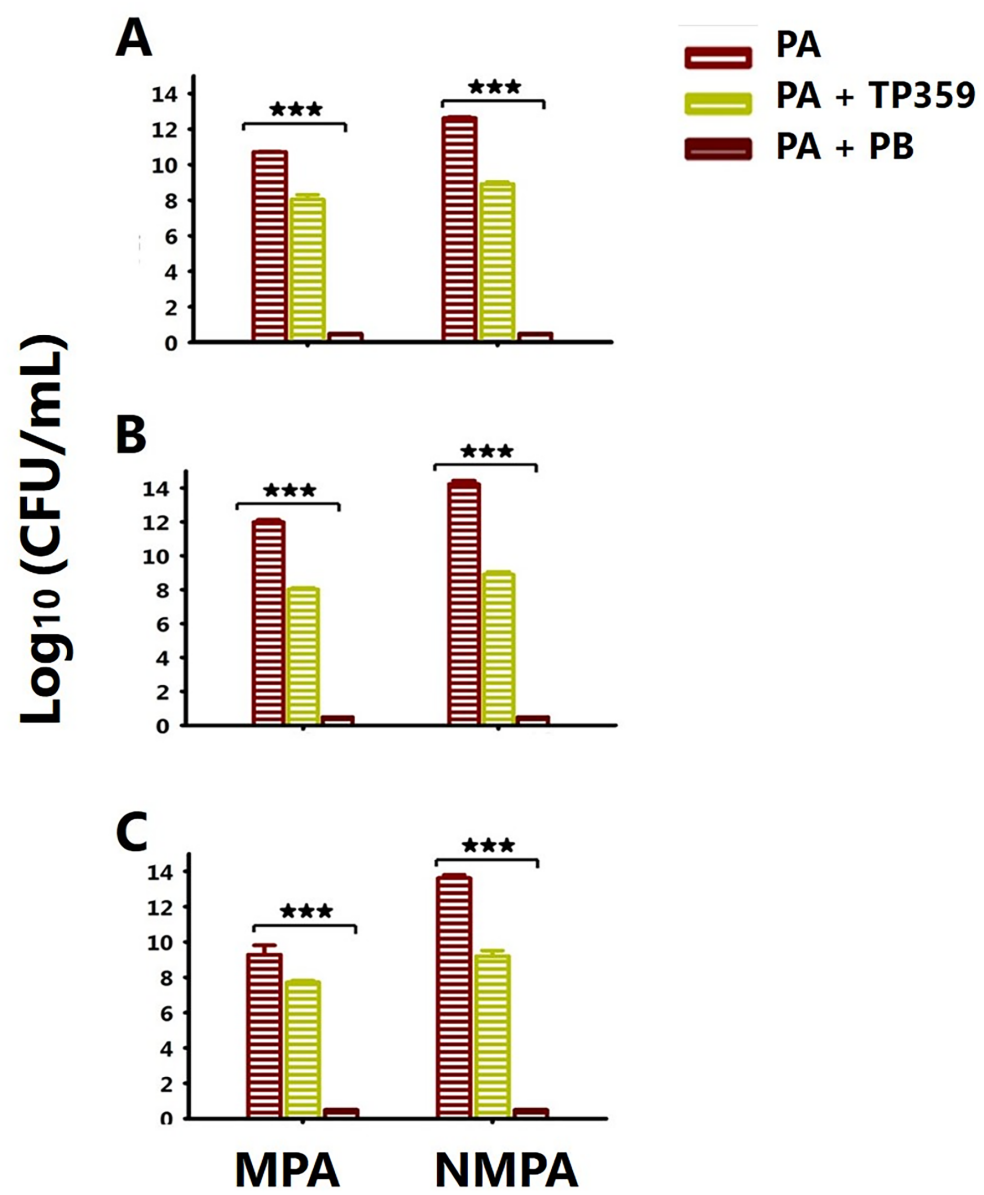
The anti-inflammatory effect of TP359 is not entirely due to its anti-microbial properties. A549 cells (1 × 10^6^ cells/well/mL) were cultured in 12-well plates overnight. A549 cells were next infected with the mucoid and non-mucoid strains of *P*. *aeruginosa* (MOIs of 100) for 4 h in the presence and absence of TP359 (100 μg/mL) using three different strategies. [A] Infecting A549 cells with bacteria and TP359 simultaneously; [B] Exposing A549 cells to TP359 for 1 h, before bacterial infection; and [C] Infecting A549 cells with bacteria for 1 h, before exposing them to TP359. Culture supernatants were collected and colony count assay was done by serial dilution on Luria agar. Each bar represents the mean ± SD of samples run in triplicates. The data are representative of three separate experiments. Statistical analysis was carried out using the one-way ANOVA. The number of asterisk was based on the degree of significance such as *P < 0.05, **P < 0.01 and ***P <0.001.

Given an already established anti-microbial effect of TP359 on *P*. *aeruginosa in vitro*, we next assessed its anti-microbial effect on *P*. *aeruginosa*-infected A549 cells by quantifying the bacterial load after a 4 h infection time-point using all three strategies as described above (Fig 4). Colony count analysis showed a significant difference (*P* < 0.001) between the TP359 treated- and untreated-infected cells. However, there was a marked difference between the positive PB control and the TP359-treated cells. PB caused complete bacteria cell death; conversely TP359 did not completely eradicate all bacteria (Fig 4A, 4B and 4C). Thus, the observed production of IL-6 and IL-8 in the PB-treated cultures (Figs 2 and 3) suggests stimulation of these cytokines potentially by dead bacteria or other unexplained/unknown mechanisms. These findings suggest that the anti-inflammatory property of the TP359 peptide may not be due entirely to its anti-microbial effect, but alternatively to other mechanisms.

### *P*. *aeruginosa* FLA is the major agonist triggering pro-inflammatory cytokines in infected A549 cells

**Fig 5:**
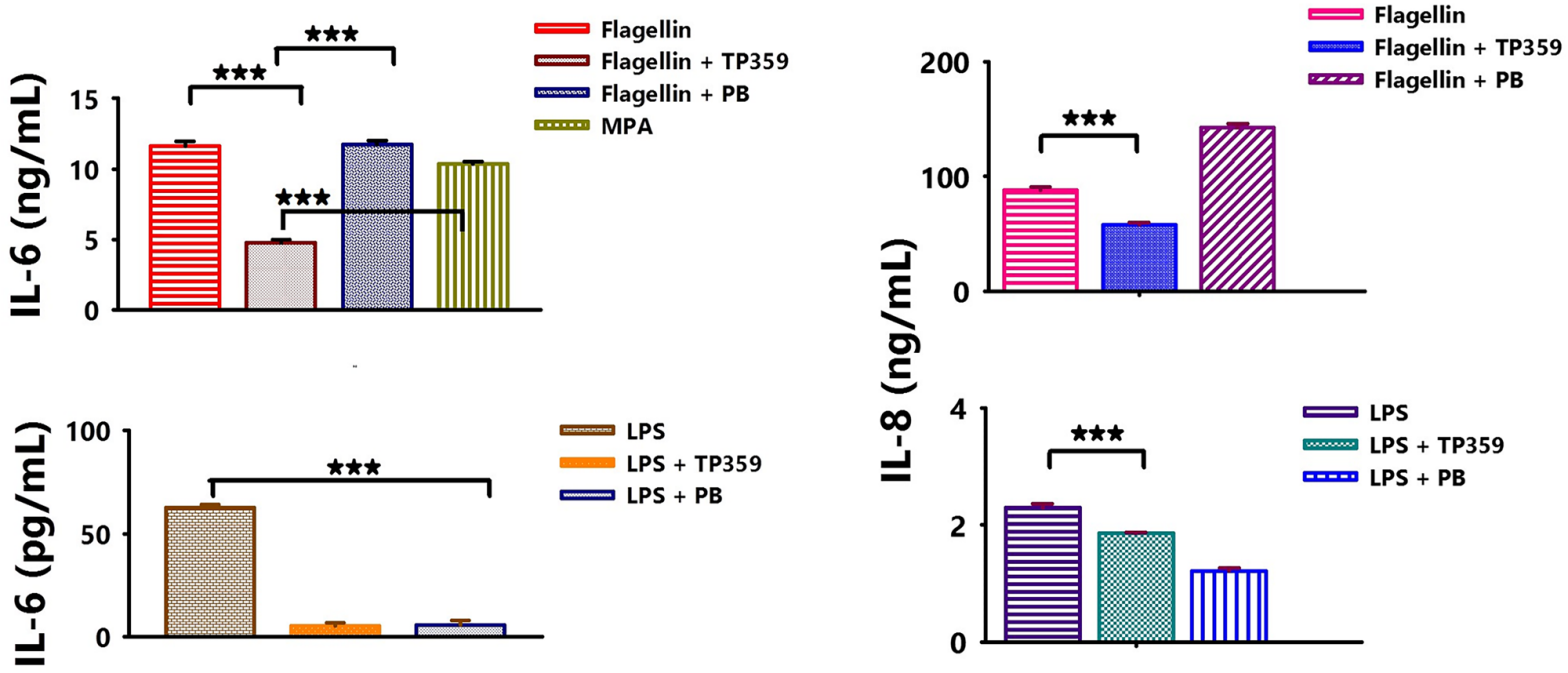
TP359 reduces the production of pro-inflammatory cytokines by A549 cells stimulated with FLA and LPS. A549 cells (1 × 10^6^/well) were cultured in 12-well plates overnight followed by stimulation with pre-determined optimum concentrations of FLA (100 ng/mL) or LPS (1 μg/mL) for 4 h in the presence or absence of TP359. Positive controls consisted of A549 cells exposed to live *P*. *aeruginosa* (MOI of 100) with or without treatment with polymyxin (PB) at 50 μg/mL. Unstimulated A549 cells served as the negative control. [A] and [B] are IL-6 and IL-8 concentration for FLA treated cells, respectively, while [C] and [D] are IL-6 and IL-8 concentration for LPS treated cells, respectively. Cell-free supernatants were collected and cytokine concentrations were measured by specific ELISAs. Each bar represents the mean ± SD of samples run in triplicates and each is representative of three independent experiments. Statistical analysis was carried out using the one-way ANOVA. Asterisk indicates significant difference *P < 0.05, **P < 0.01 and ***P < 0.001.

*P*. *aeruginosa* possesses numerous PAMPS which enable host recognition and eradication of the organism [3, 24]. TLRs are part of the host immune system, which respond to PAMPS, and the most important receptors in the *P*. *aeruginosa*-epithelial cell interaction are TLR4 and TLR5, which recognize LPS and FLA [25], respectively and participate in innate immune responses. Therefore, we wanted to identify the major PAMP responsible for induction of pro-inflammatory cytokines in our *P*. *aeruginosa* lung infection model along with examining the anti-inflammatory effect of TP359 on cytokine levels. Of surprise, *P*. *aeruginosa* FLA secreted significantly more (*P* < 0.001) IL-6 as compared to its LPS (Fig 5). The mucoid *P*. *aeruginosa* strain secreted equivalent levels of IL-6 as FLA, suggesting that FLA is the major moiety of *P*. *aeruginosa* triggering inflammatory mediators by A549 cells.

TP359 also comparatively reduced the levels of IL-6 and IL-8 (Fig 5A and 5B) in A549 cells exposed to FLA or LPS as observed for live *P*. *aeruginosa*, suggesting its potential anti-inflammatory effect on their putative receptors, TLR5 and TLR4, respectively. Together, these results further validate that the anti-inflammatory effect of TP359 is not entirely due to its anti-microbial properties.

### Neutralization of TLR4 and TLR5 inhibits secretions of IL-6 and IL-8 by stimulated A549 cells

**Fig 6:**
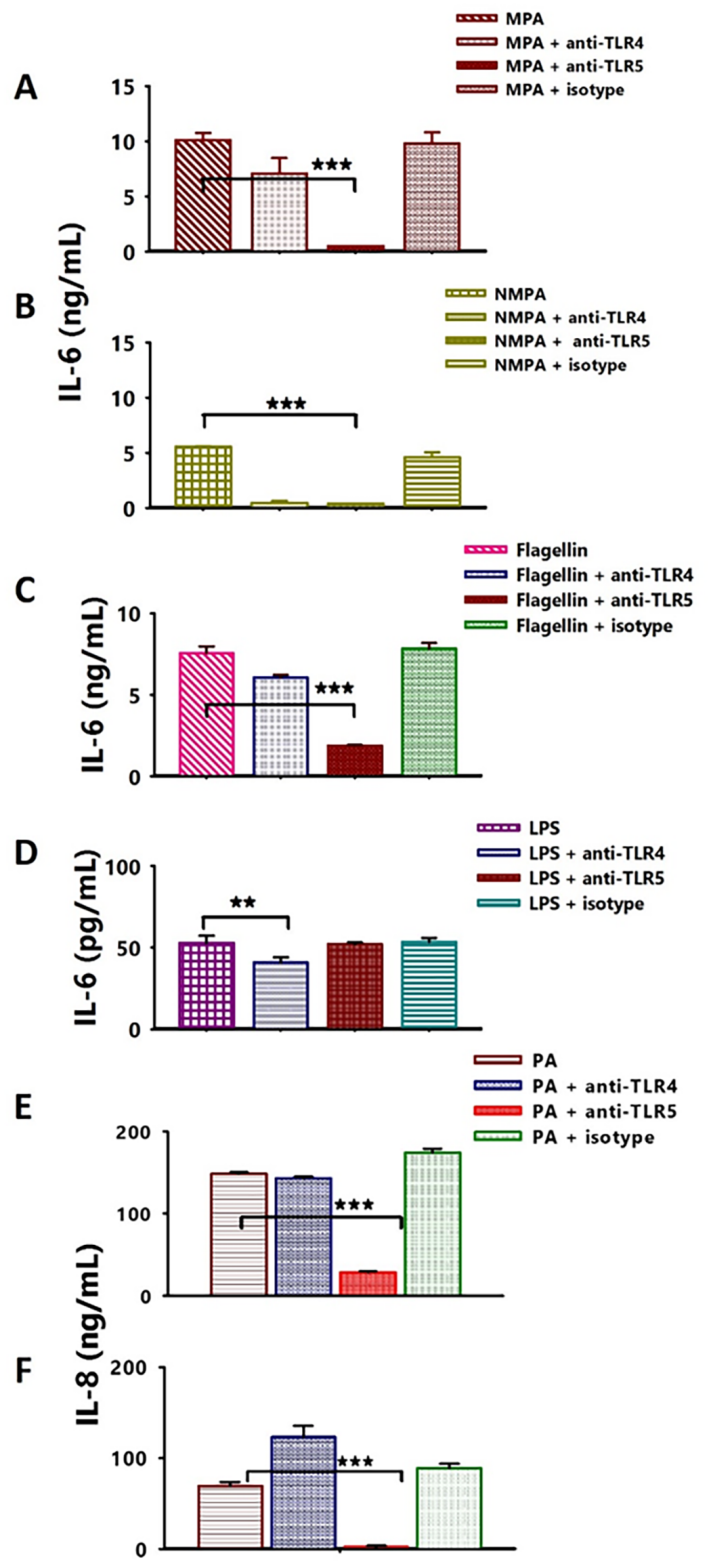
Neutralization of TLR4 and TLR5 reduces the secretions of cytokines by A549 stimulated cells. A549 cells (1 × 10^6^ cells/well) were cultured in 12-well plates for 24 h, followed by exposure to optimum concentrations of anti-TLR4 (10 μg/mL), anti-TLR5 (10 μg/mL) and their isotype controls (10 μg/mL) for an additional 24 h. *P*. *aeruginosa* (MOI of 100), FLA (100 ng/mL) and LPS (1 μg/mL) were then added and incubated for an additional 4 h followed by collection of cell-and bacteria-free culture supernatants to quantify cytokines using specific ELISAs. Asterisk indicates significant difference *P < 0.05, **P < 0.01 and *** *P* < 0.001 and P values were calculated by use of one way ANOVA Each bar represents the mean ± SD of samples run in triplicates. The data are representative of two separate experiments. [A] and [B] are IL-6 concentrations in mucoid and non-mucoid *P*. *aeruginosa*-treated A549 cells respectively, [C] and [D] are IL-6 concentrations in FLA- and LPS-treated A549 cells respectively, [E] and [F] are IL-8 concentration in mucoid and non-mucoid *P*. *aeruginosa* treated A549 cells respectively. Each bar represents the mean ± SD of samples run in triplicates and the data are representative of three separate experiments. Statistical analysis was carried out using the one-way ANOVA. Asterisk indicates significant difference *P < 0.05, **P < 0.01 and ***P < 0.001.

Given that TP359 suppressed IL-6 and IL-8 production levels in FLA- and LPS-stimulated A549 cells, we next discerned the TLR pathway that is involved in this phenomenon. A549 cells were first exposed to either anti-TLR4, anti-TLR5 and their isotype control blocking antibodies overnight, after which they were exposed to bacteria for 4 h. Positive controls consisted of anti-TLR4 or TLR5- treated A549 cells stimulated with LPS or FLA (Fig 6B), respectively. Anti-TLR-treated A549 cells exposed to bacteria secreted less IL-6 and IL-8 (Fig 6C), when compared to the untreated controls (Fig 6A). Blocking of TLR5 in A549 cells exposed to the mucoid *P*. *aeruginosa* resulted in significantly (*P* < 0.001) reduced IL-6 secretion, compared to blocking with its TLR4 counterpart (Fig 6A). Surprisingly, blocking of TLR4 and TLR5 in non-mucoid-A549-infected-cells, resulted in similar reduced IL-6 levels (Fig 6A), probably due to its outer membrane being more exposed than that of the mucoid, which is covered by alginate [9]. These results showed that TLR4 and, more specifically, TLR5 are the major receptors involved in the response of A549 cells to *P*. *aeruginosa* infection. Additionally, given that TLR4 and TLR5 recognize LPS and FLA, respectively, suppression of pro-inflammatory mediators by neutralization of these receptors proved that they are essential for mediating inflammation in infected A549 cells and for the anti-inflammatory effect of TP359.

### *P*. *aeruginosa* induces the secretion of pro-inflammatory cytokines via the p38 MAPK and JNK pathways

**Fig 7:**
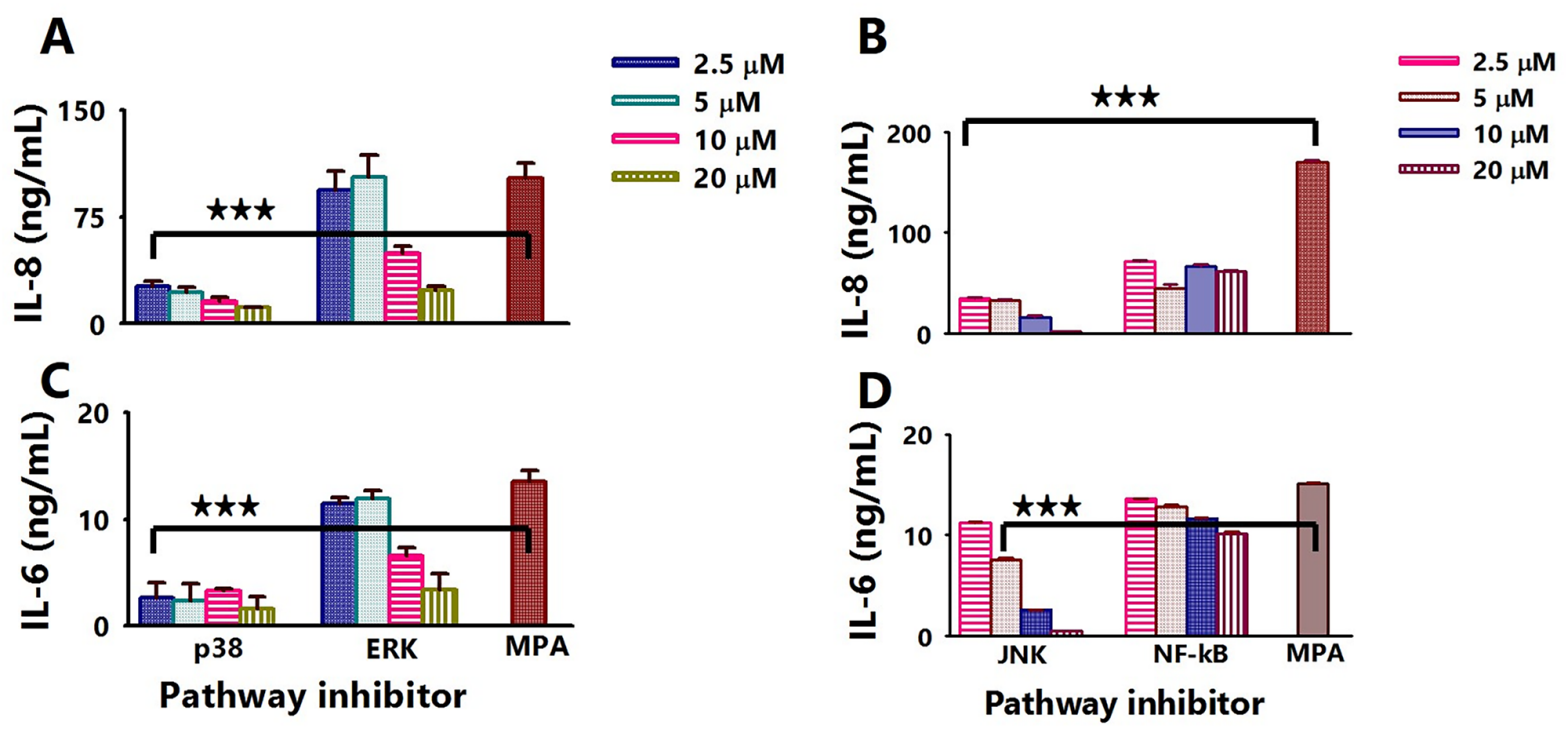
*P*. *aeruginosa* induces the secretion of pro-inflammatory cytokines via the p38 MAPK and JNK pathways. A549 cells (1 × 10^6^ cells/well) were cultured in 12-well plates for 24 h, and then exposed for 24 h to varying concentrations (2.5–20 μM) of pathway specific inhibitors: p38 MAPK (SB203580), ERK U0126), JNK (JNK-IN-8) and NF-κB (activation inhibitor IV). After 24 h incubation, bacterial strains of *P*. *aeruginosa* (MOIs of 100) were added and incubated for 4 h followed by collection of cell- and bacteria-free supernatants for quantification of cytokines using specific ELISAs. [A] and [C] are IL-8 and IL-6 concentrations, respectively in cultures with p38 and ERK inhibitors; [B] and [D] are IL-8 and IL-6 concentrations in supernatants with JNK and NF-kB inhibitors. Each bar represents the mean ± SD of samples run in triplicates with data being representative of three separate experiments. Statistical analysis was carried out using the one-way ANOVA. Asterisk indicates significant difference *P < 0.05, **P < 0.01 and ***P < 0.001.

To establish the pathway used by *P*. *aeruginosa* to induce inflammation, A549 cells were exposed overnight to various concentrations (2.5–20 μM) of pathway-specific inhibitors for p38 MAPK, JNK, ERK and NF-kB, followed by exposure to bacteria. Our results show that both IL-6 and IL-8 secretion levels were inhibited in *P*. *aeruginosa*-infected A549 cells treated with all pathway inhibitors in a dose-dependent manner (Fig 7). Specific inhibition of p38 caused the most significant (*P* < 0.001) down-regulation of IL-8 and IL-6 expression (Fig 7A) where all concentrations of p38 inhibitor resulted in equal inhibition of these cytokines. This finding indicates that the concentration of inhibitors is not directly correlated with its ability to inhibit expression of inflammatory mediators, thereby suggesting the significance of the p38 pathway in their expression. Inhibition of the ERK pathway resulted in significant inhibition in the expression of inflammatory mediators only at the 10 and 20 μM concentrations (Fig 7A) whereas, inhibition of JNK (Fig 7B) increased as the concentration of inhibitor increased, indicating that inhibition is directly correlated with its concentration. Surprisingly, NF-kB inhibitor did not show as much inhibition of cytokine expression, perhaps due to the inhibitor and/or concentration used in the study. Our pathway-specific inhibition studies show that the MAPK pathways, especially p38, are primarily necessary for *P*. *aeruginosa* to induce pro-inflammatory cytokines in A549 lung cells.

### TP359 down-regulates *P*. *aeruginosa* and FLA phosphorylation of p38 MAPK

**Fig 8:**
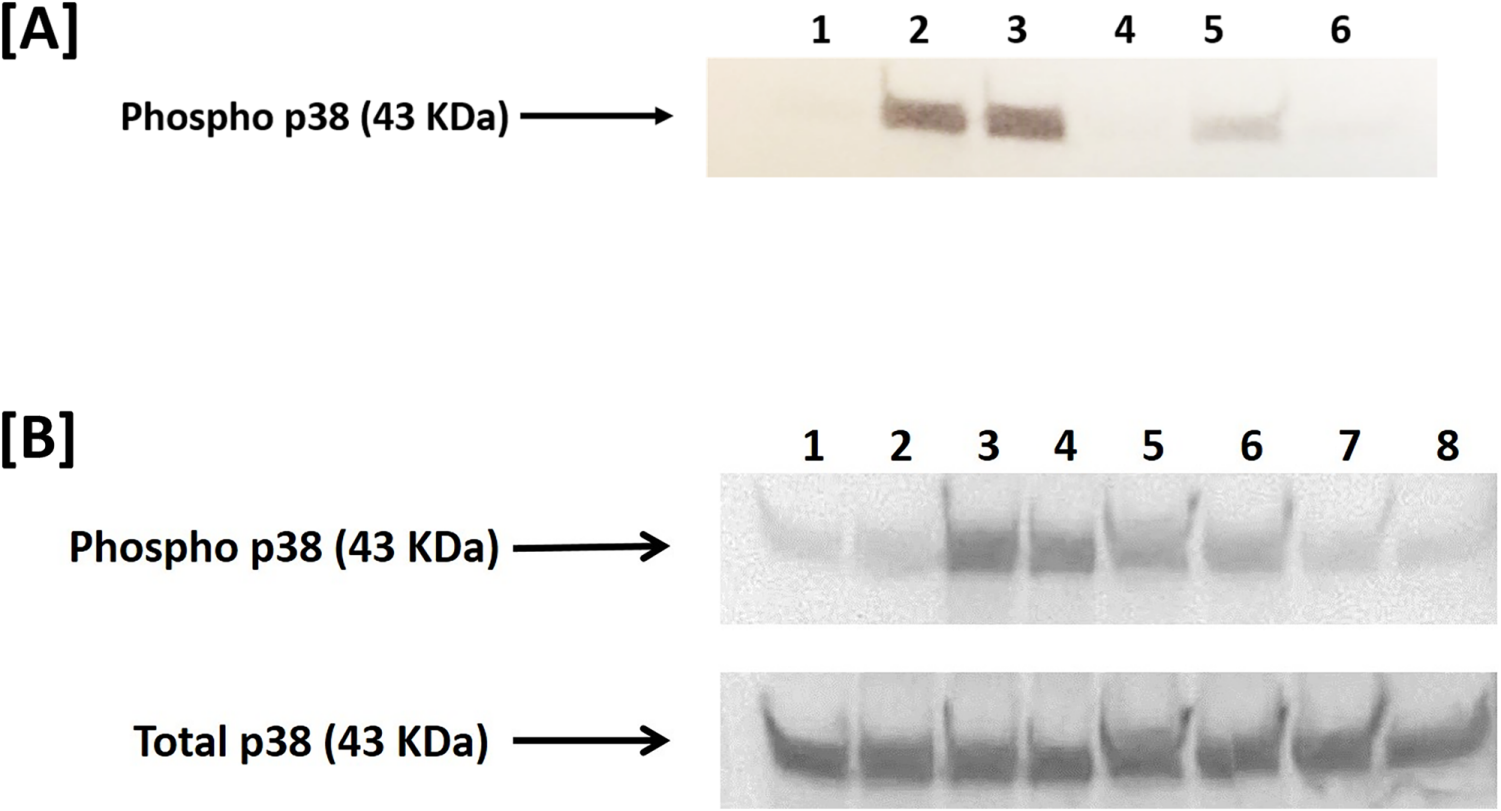
TP359 down-regulates the phosphorylation of p38 MAPK in A549 lung cells exposed to *P*. *aeruginosa* and FLA. A549 (5 × 10^6^ cells/well) were seeded in 60 mm × 15 mm cell-culture dishes and exposed to mucoid *P*. *aeruginosa* (5 × 10^8^ CFU/mL), FLA (100 ng/mL) or LPS (1 μg/mL). Protein lysates were collected at different time-points (15, 30 and 60 mins) after cell exposure and subjected to SDS-PAGE as indicated in the materials and methods section. The presence of total p38 (internal control) and phosphorylated p38 (Phospho-p38) proteins was determined by western blotting as described in the materials and methods section. The 43 KDa phospho-p38 and total p38 proteins were determined from known positive controls and biotinylated ladder. Shown in blot [A] are bands of intensity for phospho-p38 at the different time-points for A549 cells exposed to *P*. *aeruginosa*: (1) Cells alone, 60 min; (2) Infected cells, 60 min; (3) Infected cells, 30 min; (4) Cells alone, 30 min; (5) Infected cells, 15 min; (6) Cells alone, 15 min. Shown in blot [B] are bands intensity for phospho-p38 and total p38 for A549 cells treated with bacteria, FLA or LPS in the presence and absence of TP359: (1) Cells alone, (2) Cells + TP359, (3) Cells + bacteria, (4) Cells + bacteria + TP359, (5) Cells + FLA, (6) Cells + FLA + TP359, (7) Cells + LPS, (8) Cells + LPS + TP359.

Since our above results and other studies have shown the p38 MAPK signaling pathway meditates response to *P*. *aeruginosa* [26, 27], we further investigated if this pathway may be used by TP359 to exert its anti-inflammatory effect in lung cells infected with *P*. *aeruginosa* and FLA. We first established that indeed *P*. *aeruginosa* could induce the phosphorylation of p38 MAPK in human A549 lung cells for the production of its inflammatory mediators. Our time-kinetics experiment shows that *P*. *aeruginosa* infected human A549 lung cells did induce the phosphorylation of p38, with the highest phosphorylation occurring at 60 min (Fig 8A). However, in the presence of TP359, the phosphorylation of p38 was reduced as indicated by the reduced band intensity [Fig 8B, Lane 4]. Similarly, FLA induced the phosphorylation of p38 at 60 min of stimulation, which was also reduced in the presence of TP359 (Fig 8B, Lane 6). Overall, our results show TP359 mediates its anti-inflammatory effect via the p38 MAPK pathway by its ability to modulate the phosphorylation of p38 in both *P*. *aeruginosa-* and FLA- stimulated human lung cells.

### Proposed TP359 anti-inflammatory mode of action in lung cells

**Fig 9:**
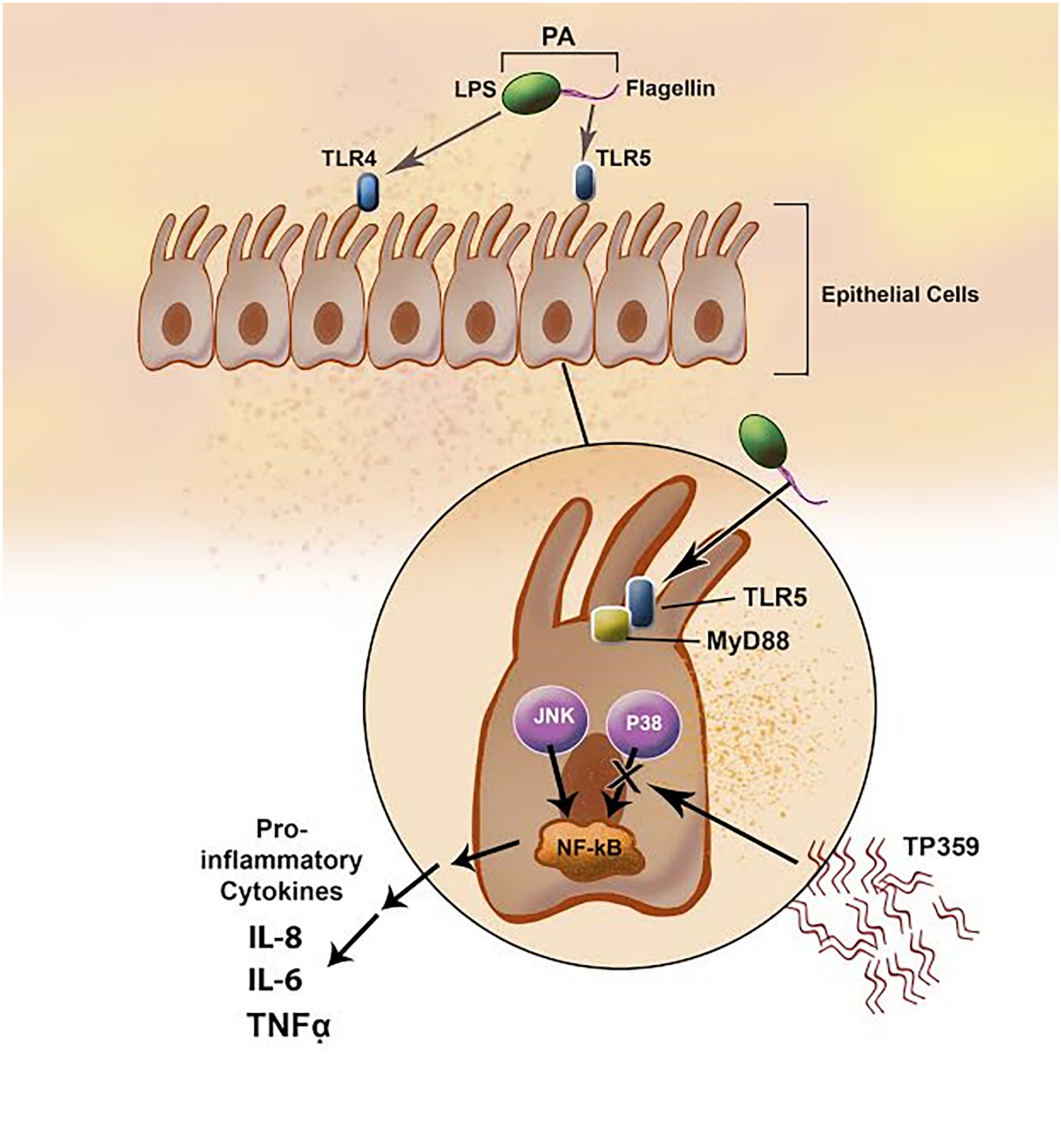
Proposed mechanism of action of TLR5 recognition of flagellin and TP359 attenuation of inflammation in human A549 lung cells. The TLR5 signaling cascade on the cell surface is stimulated by the binding of bacterial flagellum which is composed of flagellin. Flagellin-stimulated TLR5 recruits the adapter protein, MyD88, which activates MAP kinases, p38, JNK, and the transcription factor NF-κB. NF-κB further induces downstream pro-inflammatory pathways resulting in the secretion of pro-inflammatory cytokines such as IL-8, IL-6 and TNFα. TP359 attenuated inflammation in human A549 lung cells exposed to *P*. *aeruginosa* and flagellin by down-regulating the phosphorylation of the p38 MAPK pathway or blocking the binding of flagellin to TLR5 receptor, thereby inhibiting the expression of pro-inflammatory cytokines.

Based on results obtained in this study, we proposed that *P*. *aeruginosa* induces inflammation in human A549 epithelial cells by the interaction of the flagella with the TLR5 dependent p38 signaling pathway. TP359 then attenuates inflammation in human A549 lungs cells exposed to *P*. *aeruginosa* and flagella by the down-regulation of the activation of the p38 MAPK pathway thereby inhibiting the secretion of pro-inflammatory cytokines (Fig 9)

## Discussion

*P*. *aeruginosa* attaches to the epithelial cells of the respiratory tract, causing robust inflammatory responses, particularly in CF patients. The persistent *P*. *aeruginosa*-induced inflammatory responses, coupled with the inability of the host to modulate the response, result in progressive deterioration of lung function [28]. Due to the severity of *P*. *aeruginosa* infection and its induction of inflammation, there is a need for new anti-microbial and anti-inflammatory therapeutics. AMPs are vastly studied for their anti-microbial activity and considered as the next generation therapeutics, due to their broad-spectrum activities [21, 29]. Their anti-inflammatory actions are documented [30–33] together with their immune-modulatory properties. For example, the role of the human cathelicidin cationic anti-microbial peptide, LL-37, has been discovered in skin inflammatory diseases, such as psoriasis and dermatitis [34]. Hence, AMPs are attractive prospective therapeutic agents due to their anti-microbial and anti-inflammatory properties [5, 35].

Recently, we showed that the proprietory peptide, TP359, had potent bactericidal effects against *P*. *aeruginosa* [21]. Here in this study, we examined whether TP359 also had an anti-inflammatory effect on pro-inflammatorycytokines produced by human A549 lung cells infected with live *P*. *aeruginosa* (non-isogenic mucoid and non-mucoid strains). Our results provided the following observations: 1) The mucoid strain induced more IL-6, IL-8 and TNFα in A549 lung cells as compared to the non-mucoid strain; 2) TP359 reduced IL-6, IL-8 and TNFα as elicited from A549 cells infected with *P*. *aeruginosa*, which was partly due to its antimicorbial effect, 3) *P*. *aeruginosa* FLA and not its LPS is the major agonist responsible for inducing pro-inflammatory cytokines in A549 cells; 4) Blocking of TLR4 and TLR5 receptors, and particularly TLR5 reduced the secretion of cytokines by infected A549 cells, 5) Pathway inhibition studies revealed that *P*. *aeruginosa* induced the secretion of cytokines mainly via the MAPK pathway, especially p38 MAPK; and 6) TP359 inhibited the ability of *P*. *aeruginosa* and FLA to phosphorylate p38 MAPK for the production of pro-inflammatory cytokines by A549 lung cells.

Our results revealed that the mucoid strain of *P*. *aeruginosa* induced more pro-inflammatory cytokines (IL-6, IL-8 and TNFα) relative to the non-mucoid strain, suggesting the stimulatory capacity of the mucoid strain. Another study [36] reported that the mucoid phenotype and not two non-mucoid phenotypes of *P*. *aerugnosa* induced the anti-microbial factor, hβD-2, by airway epithelial cells. These findings, along with ours, suggest that the mucoid strain is more likely to stimulate respiratory epithelial cells than their non-mucoid counterparts. However, earlier findings [7] are contradictory, where no differences were observed in cytokine levels between isogenic mucoid and non-mucoid *P*. *aeruginosa* in a mouse model of concurrent respiratory infection. With the difference between mucoid and non-mucoid strains being the synthesis of excess alginate, surprisingly, alginate alone is incapable of trigerring immune responses [37].

We observed that TP359 reduced the production levels of IL-6, IL-8 and TNFα in pulmonary lung cells exposed to live *P*. *aeruginosa*; these cytokines are physiologically elevated in the airways of CF patients [38, 39]. This inflammatory response initiated by the airway epithelial cells is due to cells responding to bacteria by influx of neutrophils to the infected site [2]. The anti-inflammatory property of TP359 may also be partially attributed to its anti-microbial activity, because TP359 retained its anti-microbial effect on the A549-*P*. *aeruginosa* infected cells with a 3–4 log reduction in live-*P*. *aeruginosa*. However, the anti-inflammatory property attributed to the anti-microbial effect is only partial, due to the fact that the number of live- *P*. *aeruginosa* is inversely proportional to the significantly reduced inflammatory response, thereby indicating other factors were involved to reduce the lung inflammation.

Many AMPs have been reported to possess anti-inflammatory properites [40–43], but their mechanism of action remains uncertain. However, due to their established anti-microbial mechanisms of action, their anti-inflammatory mode of action has been, understandably, attriubuted to their ability to block LPS recognition or to scavenging. LPS and FLA, especially FLA of *P*. *aeruginosa* are the dominant PAMPs that trigger the host immune system upon infection. Interestingly, alginate and FLA are inversely regulated [44], suggesting the mucoid phenotype may still possess the FLA protein. Our results demonstrate that the anti-inflammatory action of TP359 is not due to LPS recognition, as also underscored with our recent study [21]. We proved that unlike most AMPs, whose anti-microbial mode of action is entirely attributed to the ability of the positively charged peptide to interact with the negatively charged LPS, TP359 still down-regulated the expression of membrane biogenesis genes.

It has been reported that *P*. *aeruginosa* FLA and LPS are adequate to trigger host immune responses [3]. Similarly in our study, we observed that both of these agonists stimulated the production of pro-inflamamtory cytokines, but with FLA and not LPS being the predominant stimulant. Thus, LPS is not the major inducer of inflammation by *P*. *aeruginosa* in the airways, which is consistent with other reports, where *P*. *aeruginosa* FLA and not its LPS, or *E*.*coli* LPS stimulate human airway epithelial cells [45]. Another study demonstrated that the activation of airway epithelial innate responses by *P*. *aeruginosa* is dependent on FLA expression [46–48], suggesting that FLA is the major component that stimulates cellular responses in *P*. *aeruginosa* infection. The reduced stimulation by LPS may be a result of a change in downstrean recognition by TLR4. Of significance was the obsevation that TP359 also reduced the secretion of pro-inflamamtory cytokines in A549 cells exposed to *P*. *aeruginosa* FLA and LPS agonists.

The TLR5 signaling cascade on the cell surface is stimulated by the binding of bacterial flagellum, which is composed of globular protein, FLA. FLA-stimulated TLR5 recruits the adapter protein, MyD88, which activates MAP kinases, p38, JNK, and the transcription factor NF-κB. NF-κB further induces downstream pro-inflammatory pathway resulting in activation of innate immune responses against flagellated bacteria [49–50] by the secretion of pro-inflammatory cytokines such as IL-8, IL-6 and TNFα. Neutralization of the TLR4 and TLR5 receptors resulted in reduced expression of pro-inflammatory cytokines, thereby indicating that both receptors, particularly TLR5 is essential for bacterial infection-induced inflammatory responses, which further supports our findings that FLA is the major stimulator of immune responses in the human-A549-*P*. *aeruginosa* infected cells

The production of cytokines such as IL-6, IL-8 and TNFα by *P*. *aeruginosa* has been reported to occur through the NF-kB and MAPK pathways [51]. Secretion of IL-8 in CF airway epithelial cells, reportedly, was not due to the increase in NF-kB-dependent IL-8 promoter transcription, but to p38 MAPK and ERK [52].In our study, inhibition of p38 MAPK remarkably attenuated the secretion of IL-6 and IL-8, suggesting that p38 MAPK may be essential for inflammatory responses in the A549 cell response to *P*.*aeruginosa*. However, contradictory reports exist concerning the role of p38 MAPK in *P*. *aeruginosa* signaling [44]. In that report, p38 did not translocate into the nucleus, to stimulate NF-kB, suggesting that p38 may unlikely be involved in direct phosphorylation of nucleus-located transcription factor. Another study suggested that p38 MAPK may activate the NF-kB signaling pathway through the phosphorylation of p65 [52]. This same study revealed that the inhibition of JNK significantly attenuated the secretion of IL-6 and IL-8, corroborating our results and underscoring the significance of JNK in *P*. *aeruginosa*-induced inflammatory response as well as other possible routes for NF-kB activation. It is surprising to note that inhibition of NF-kB and ERK pathway did not significantly inhibit the secretion of IL-6 and IL-8 when compared to p38 and JNK. Furthermore, phosphorylation of p38 by *P*. *aeruginosa* in human A549 lung cells in this study (Fig 8) showed that it utilizes this pathway for producing its concomitant pro-inflammatory cytokines. TP359 inhibited the ability for *P*. *aeruginosa* and FLA to phosphorylate p38 MAPK in A549 cells, suggesting possibly its attenuation of the produced cytokines.

Our results suggest that the ensuing TP359 regulation of pro-inflammatory cytokines was undoubtedly due to blocking the interaction of TLR5 with its FLA agonist and preventing down-stream activation of signaling pathways. This is underscored by the observation that neutralization of the TLR5 receptor resulted in reduced secretion of pro-inflammatory cytokines as elicited by FLA coupled with down-regulating the phosphorylation of the p38 MAPK pathway by FLA, which would attenuate the secretion of pro-inflammatory cytokines. Concomitantly, the anti-microbial effect of TP359 against *P*. *aeruginosa* in infected lung cells may also be a contributory factor in its anti-inflammatory mechanism of action.

Taken together, our findings suggest that *P*. *aeruginosa* induced the secretion of pro-inflammatory cytokines through the upstream and downstream TLR5-dependent MAPK signaling pathway and possible interaction of TP359 with the p38 MAPK signaling pathways. This may serve as a critical therapeutic target for modulation of inflammation in human lung airways. Thus, we propose that TP359, which exhibits both anti-microbial and anti-inflammatory activities, may be applicable as a potential therapeutic molecule against *P*. *aeruginosa* infection.
